# The ISWI chromatin remodelling factor NURF is not required for mitotic male X chromosome organisation

**DOI:** 10.17912/micropub.biology.000360

**Published:** 2021-01-26

**Authors:** So Yeon Kwon, Boyun Jang, Paul Badenhorst

**Affiliations:** 1 Birmingham Centre for Genome Biology and Institute of Cancer and Genomic Sciences, College of Medical and Dental Sciences, University of Birmingham, Edgbaston, United Kingdom

## Abstract

The nucleosome remodelling factor (NURF) is an ISWI-class ATP-dependent chromatin remodeling enzyme required both for gene expression and higher order chromatin organisation*. *NURF binds to histone modifications that decorate the *Drosophila* polytene male X chromosome and is required to maintain correct organisation of this chromosome. NURF mutants exhibit distorted and decondensed polytene male X chromosomes dependent on the presence of the male-specific lethal (MSL) complex. Here we tested whether mitotic chromosomes similarly require NURF to maintain correct morphology. Surprisingly, although the MSL complex remains associated with mitotic male X chromosomes, NURF is not required to maintain morphology. While the ISWI subunit of NURF is known to remain associated with mitotic chromosomes we show that the NURF specificity subunit Nurf301/BPTF dissociates from chromatin during both *Drosophila* and human mitosis, further illuminating that NURF is dispensable for mitotic chromosome organisation.

**Figure 1. The chromatin remodelling factor NURF is not required for mitotic chromosome cohesion, condensation or X chromosome morphology:**
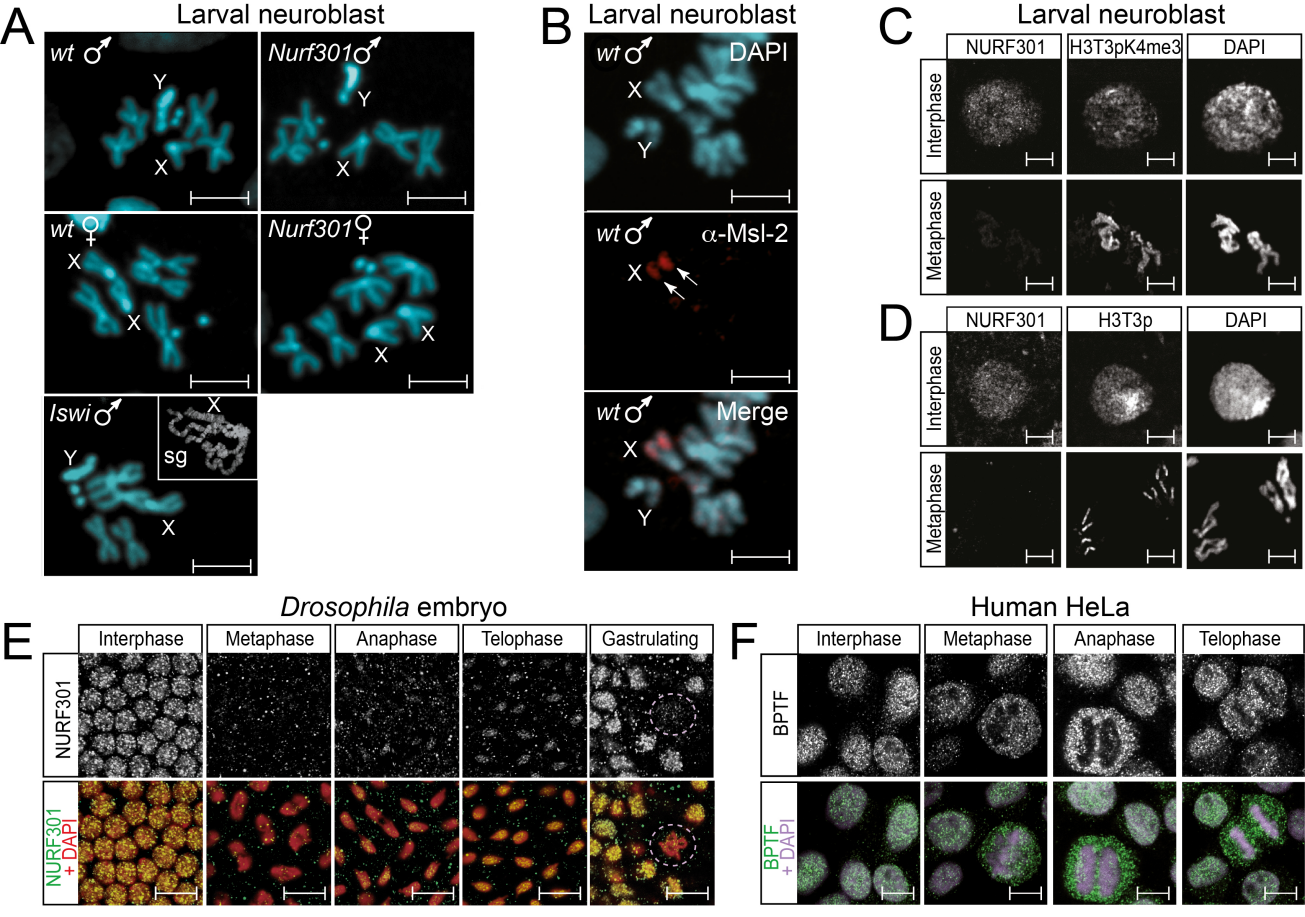
**A)** Mutation of either the NURF specificity subunit *Nurf301* or the NURF catalytic subunit *Iswi* does not disrupt male X chromosome morphology or result in loss of sister chromatid cohesion. Representative neuroblast mitotic chromosome spreads from control (*w^1118^*), *Nurf301^2^* homozygous mutant and *Iswi^1^/Iswi^2^* transheterozygous mutant male and female larval brain squashes are presented. X, X chromosome, Y, Y chromosome. Inset (sg) indicates the bloated male polytene X chromosome phenotype observed in salivary glands from the same animal used for mitotic chromosome preparations. **B)** Antibody staining using anti-Msl-2 antibodies reveals that components of the dosage compensation complex remain associated with mitotic male X-chromosomes (arrows). **C-D)** In contrast Nurf301 is not associated with mitotic chromosomes as revealed by double staining with anti-Nurf301 antibodies and either **C)** anti-H3T3pK4me3 or **D)** anti-H3T3phistone modification antibodies as controls. Interphase nuclei (which display chromatin-associated Nurf301) and mitotic chromosomes of *Drosophila* 3^rd^ instar larval neuroblasts are presented. Scalebars in **A-D** represent 5 µm. **E)** Anti-Nurf301 staining (green in merge) of *w^1118^* embryos confirms that Nurf301 is localised to chromatin in embryos during interphase, but is not chromatin-bound during metaphase, beginning to re-localise to chromatin during anaphase with levels rising during telophase. This is observed both in blastoderm stage and gastrulating embryos where dividing nuclei are indicated (lilac ovals). DNA is revealed by DAPI staining (red in merge). **F**) Delocalisation of the NURF signature subunit from mitotic chromatin is also observed in human cells where anti-BPTF staining shows NURF is not chromatin bound in mitotic HeLa cells. Scalebars in **E,F** represent 10 µm.

## Description

*Drosophila* NURF is an ISWI-containing chromatin remodeling complex that catalyzes ATP-dependent nucleosome sliding. By sliding nucleosomes, NURF can alter chromatin dynamics to control both transcription and genome organisation. NURF is composed of four subunits including the catalytic subunit Iswi and a large, highly conserved, NURF-specific scaffold subunit. In *Drosophila* this large subunit is Nurf301 (also known as Enhancer of bithorax (E(bx)) or CG32346, FlyBase ID:FBgn0000541) in humans bromodomain and PHD finger transcription factor (BPTF)) (Barak *et al.*, 2003; Xiao *et al.*, 2001). Nurf301/BPTF contains domains that have the potential to recognize specific histone modifications including a C-terminal bromodomain that recognizes histone H4 acetylated at lysine position 16 (H4K16Ac) (Kwon *et al.*, 2009; Ruthenberg *et al.*, 2011). H4K16Ac is enriched on the Drosophila male X chromosome as a result of the actions of the male-specific lethal (MSL) complex (reviewed in Samata and Akhtar, 2018) and in addition to being bound by Nurf301 can influence the activity of the Iswi subunit of NURF (Clapier *et al.*, 2001; Corona *et al.* 2002).

Mutations in NURF subunits exhibit striking disruption of male polytene X chromosomes. This includes a characteristic decondensation or “bloating” of male polytene X chromosomes, showing that NURF functions to organise extensive regions of the male polytene X chromosome. Polytene chromosomes are the large chromosomes of the giant nuclei of salivary glands (reviewed in Zhimulev and Koryakov (2009)) that result from as many as ten rounds of endoreplication, where DNA is replicated without intervening mitosis (Hammond and Laird, 1985). The large number (1024C) of resultant sister chromatids and homologous chromosomes are held together generating single large chromosomes (Urata *et al.*, 1995) that display features of interphase chromosome organisation including topologically associated domains (Eagen *et al.*, 2015). Previous work has shown that the NURF mutant male polytene X chromosome phenotype is dependent on the presence of the MSL complex and that NURF functions on the male polytene X chromosome by antagonising the MSL complex (Badenhorst *et al.* 2002, Bai *et al.*, 2007; Deuring *et al.* 2000).

Here we investigated whether this requirement is a general feature of all chromosome states. In particular we sought to establish whether NURF is also required for organisation of mitotic male X chromosomes. We examined morphology of mitotic chromosomes in neuroblasts of third instar *Drosophila* larvae from both control larvae (*w^1118^*) and *Nurf301* or *Iswi* mutant larvae. Mitotic chromosomes were prepared from both male and female larvae. As shown in **Fig. 1A** no differences in male X chromosome morphology relative to wild-type were detected in either male *Nurf301* or *Iswi* mutant animals. As control the characteristic distorted polytene male X chromosome phenotype could be observed in salivary glands from the same mutant animals used for preparation of neuroblast squashes (inset labelled sg in **Fig. 1A**).

The lack of a mitotic male X chromosome requirement for NURF could be simply due to the dissociation of the MSL complex from mitotic chromosomes. It was shown previously that the bloated NURF mutant male polytene X chromosome is dependent on the presence of the MSL complex. MSL complex mutants or deletion of high affinity MSL complex binding sites are able to suppress the NURF mutant male polytene X chromosome phenotype (Bai *et al.*, 2007; Corona *et al.*, 2002). However, immunofluorescence microscopy of mitotic chromosomes using anti-Msl-2 antibodies detected continued presence of Msl-2 on mitotic chromosomes (**Fig. 1B**). Thus, even though components of the MSL complex remain associated with mitotic chromosomes, NURF is dispensable for correct mitotic male X chromosome morphology.

Further inspection of neuroblast squashes indicated no discernible defects in organisation of mitotic autosomes in male or female *Nurf301* or *Iswi* mutants (**Fig. 1A)**. In addition, although mammalian ISWI complexes have been implicated in cohesin loading and maintenance of chromatid cohesion (Hakimi *et al.*, 2002; Zikmund *et al.*, 2020), our analysis showed no discernible defects in sister chromatid cohesion in either male or female *Iswi* mutants (**Fig. 1A**). The lack of observable mitotic chromosome phenotypes in NURF mutants was interesting given previous analysis showing that Iswi remains associated with mitotic chromosomes (Deuring *et al.*, 2000). However Iswi is the catalytic subunit of at least seven remodeling complexes (Oppikofer *et al.*, 2017). To establish whether NURF remains associated with mitotic chromatin we therefore examined chromosome association of the NURF-specific Nurf301 subunit in larval neuroblasts.

Initial anti-Nurf301 antibody staining of neuroblast squashes prepared using standard methodologies failed to detect Nurf301 at any stage of the cell cycle. However, these protocols employed acetic acid treatment to assist chromosome spreading (Pimpinelli *et al.*, 2010). We observed that acid treatment caused either the loss or masking of signal from Nurf301 and certain histone modifications and therefore modified chromosome preparation methods to omit acid treatment, deploying instead an acid-free fixation protocol developed for polytene chromosomes (Di Mario *et al.*, 2006). Interphase nuclei and mitotic chromosomes from larval neuroblasts of control third instar *Drosophila* larvae were double-immunostained with antibodies against Nurf301 as well as antibodies against histone modifications that are enriched on mitotic chromosomes: anti-histone H3 phosphorylated at threonine position 3 (H3T3p) as well as the phosphomethyl mark (H3T3pK4me3). While we could readily detect Nurf301 in interphase neuroblast nuclei, Nurf301 signal was absent from mitotic chromosomes (**Fig. 1C,D**). This was not due to inability to stain chromatin proteins on mitotic chromosomes as both the H3T3pK4me3 (**Fig. 1C**) and the H3T3p mark (**Fig. 1D**) were detected.

These data suggest that NURF is ejected from mitotic chromatin in neuroblasts. To confirm whether this is generally observed in other tissues or organisms we performed immunostaining of *Drosophila* embryos and human HeLa cells using either anti-Nurf301 or anti-BPTF antibodies. Immunostaining of *Drosophila* embryos revealed Nurf301 was predominantly localised to chromatin during interphase (**Fig. 1E**). During metaphase Nurf301 was delocalized from chromosomes, re-associating with chromatin from anaphase. In a similar manner human BPTF was detected on interphase HeLa cell chromatin but was not localised to chromosomes during mitosis (**Fig. 1F**).

Cumulatively these data indicate that NURF is ejected from mitotic chromatin and is not required for proper mitotic chromosome condensation or cohesion. Moreover, although *Nurf301* mutant male *polytene* X chromosomes are distorted and decondensed, *Nurf301* mutant male *mitotic* chromosomes are unaffected. Strikingly, while targeting of the MSL complex to polytene chromosomes is sufficient to trigger aberrant chromosome morphology in *Nurf301*mutants (either normally on the male X chromosome or when targeted to autosomes using roX transgenes (Bai *et al.*, 2007)), the MSL complex is unable to do so on when present on mitotic chromosomes. It is tempting to speculate that this reflects underlying differences in the ability of the MSL complex to promote transcription on polytene versus mitotic chromosomes. Even though recent data indicate that mitotic chromosomes are not transcriptionally silent as has previously been believed, levels of transcription are generally lower (Palozola *et al.*, 2017).

## Methods

**Preparation of *Drosophila* neuroblast mitotic chromosomes**

The *Nurf301^2^* strain and isogenic *w^1118^* control strain are as described(Badenhorst *et al.* (2002)). *Iswi^1^* and *Iswi^2^* were obtained from John Tamkun and are as described (Deuring *et al.* (2000)). All strains were raised at 22ºC. Mitotic chromosomes were prepared as described by Pimpinelli *et al.* (2010). In brief 3^rd^ instar larval brains were dissected in saline (0.7% NaCl), washed briefly in saline and transferred to hypotonic solution (0.5% Sodium Citrate) for 10 minutes. Brains were cleared in 45% acetic acid on a siliconized coverslip, fragmented using a tungsten needle and squashed onto slides between blotting paper. Slides were frozen in liquid nitrogen, coverslips removed and slides mounted in Vectashield with DAPI (Vector Laboratories). For anti-Msl-2 staining, the same protocol was followed except after freezing samples were fixed in 1% paraformaldehyde (Polysciences, 18814-10) for 10 minutes prior to two washes in PBTw buffer (1XPBS, 0.1% Tween-20). Primary antibody incubation was performed in blocking buffer (1XPBS, 0.1% Tween-20, 0.1% FCS) containing anti-Msl-2 antibodies (1:1000) overnight. Slides were washed three times in PBTw buffer, followed by secondary antibody incubation in PBTw containing Cy3-conjugated anti-Rabbit IgG (H+L) (Jackson ImmunoResearch, 1:400) for 2 hours. Slides were washed in PBTw as above, mounted in Vectashield with DAPI (Vector Laboratories) and imaged using a Zeiss LSM780 confocal microscope.

Immunostaining of mitotic chromosomes for Nurf301 and histone modifications followed the same procedure with the exception that after hypotonic treatment brains were processed using an acid-free protocol as described by DiMario *et al.* (2006). Briefly, brains were fixed in Brower’s fixation buffer (150mM PIPES, 3mM MgSO4, 1.5mM EGTA, 1.5% NP-40 (pH 6.9) containing 2% formaldehyde (Polysciences, 18814-10)) for 3 minutes, washed in PBT (1XPBS, 0.1% Triton-X100) for 2.5 minutes, transferred to 50% Glycerol for 5 minutes, fragmented and squashed onto Poly-L-lysine (Sigma, P8920) coated slides. Slides were frozen in liquid nitrogen then processed for double-immunostaining. Slides were incubated with anti-Nurf301 antibodies (1:200 in blocking buffer) overnight at 4°C then washed with PBTw three times for 10 minutes. Secondary antibody incubation was conducted using Cy3-conjugated AffiniPure Fab Fragment Goat Anti-Rabbit IgG (Jackson ImmunoResearch, 1:400) in PBTw for two hours at room temperature, slides were then washed with PBTw three times for 10 minutes and fixed in 1% formaldehyde for 10 minutes. Samples were washed in PBTw as above and incubated with unconjugated AffiniPure Fab Fragment Goat Anti-Rabbit IgG (Jackson ImmunoResearch, 1:20) in PBTw for two hours at room temperature to block the first rabbit IgG. Samples were washed with PBTw as above and the second primary antibody reaction was performed in blocking buffer overnight at 4°C. Rabbit anti-phospho-trimethyl Histone H3 (Thr3/Lys4 & Thr22/Lys23) antibody or rabbit anti-phospho-Histone H3 (Thr3) antibody were used at 1:1000 and 1:1500 respectively. Slides were washed as above and the second secondary antibody reaction performed using FITC-conjugated AffiniPure Fab Fragment Goat Anti-Rabbit IgG (Jackson ImmunoResearch, 1:400) for two hours at room temperature in the dark. Samples were washed as above, then mounted and imaged by confocal microscopy as described above.

**Fixation and permeabilization of *Drosophila* embryos**

*Drosophila* embryos were collected on apple agar collection plates and rinsed with water onto a Nitex mesh sieve to remove residual yeast. Embryos were dechorionated in 5% sodium hypochlorite solution (Fluka, FL71696) for three minutes. The embryos were transferred to a 1.5ml Eppendorf tube containing 50% n-heptane (Sigma) and 50% PEM-formaldehyde solution (0.1 M PIPES (pH 6.95), 2 mM EGTA, 1 mM MgSO4, 4% formaldehyde (Pierce)). Embryos were fixed for 25 minutes at room temperature on a rotator. The aqueous layer was removed, 1 volume of methanol was added, and the tube was vortexed for one minute to remove the vitelline membrane. The heptane/methanol phases were allowed to separate and upper phases were removed. Two washes were performed with 1 ml methanol and then with 1:1 dilution of methanol and PBTw (0.1% Tween 20 in 1XPBS). Embryos were washed with 1ml PBTw three times for five minutes on a rotator and immunostaining performed as described above.

**Huma cell culture and immunofluorescence microscopy**

HeLa cells (CCL2 isolate, RRID: CVCL_0030) were obtained from the ATTC and STR profile verified (Amelogenin: X, CSF1PO: 9,10; D13S317: 12,13.3; D16S539: 9,10; D5S818: 11,12; D7S820: 8,12; THO1: 7; TPOX: 8,12; vWA: 16,18). HeLa Cells were incubated at 37ºC with 5% CO_2_ and cultured in Dulbecco’s Modified Eagle’s Medium (DMEM) containing 10% FBS, 100U Penicillin-Streptomycin (Sigma), 1mM Glutamine (GIBCO) and 1x non-essential amino acids (Sigma). Slides were prepared for immunofluorescence microscopy as described in Kwon *et al.* (2020), blocked overnight at 4ºC in blocking buffer (1XPBS, 0.1% Tween-20, 0.1% FCS). Primary antibody incubation using anti-BPTF/FALZ antibodies (1:200) was performed in blocking buffer for 2 hours followed by three washes in PBTw buffer (1XPBS, 0.1% Tween-20). Secondary antibody incubation was performed in PBTw at room temperature for 1 hours using Cy3-conjugated AffiniPure goat anti-rabbit antibody (Jackson ImmunoResearch Cat. 111-167-003) at 1:400 dilution. Slides were washed as above, mounted using Vectashield with DAPI (Vector Laboratories) and imaged using a Zeiss LSM780 confocal microscope.

## Reagents

**Table:** 

**REAGENT OR RESOURCE**	**SOURCE**	**IDENTIFIER**
Antibodies		
Rabbit anti-phospho-trimethyl Histone H3 (Thr3/Lys4 & Thr22/Lys23) antibody	Millipore	Cat. 07-458
Rabbit anti-phospho-Histone H3 (Thr3) antibody	Millipore	Cat. 07-424
Rabbit anti-NURF301 antibody	Own laboratory	Kwon *et al.* (2016)
Donkey AffiniPure Donkey Anti-Rabbit IgG (H+L) Cy3-conjugated	Jackson ImmunoResearch	Cat. 711-165-152
Goat AffiniPure Fab Fragment Anti-Rabbit IgG, Cy3-conjugated	Jackson ImmunoResearch	Cat. 111-167-003
Goat AffiniPure Fab Fragment Anti-Rabbit IgG FITC-conjugated	Jackson ImmunoResearch	Cat. 111-097-003
Goat AffiniPure Fab Fragment Anti-Rabbit IgG	Jackson ImmunoResearch	Cat. 111-007-003
Rabbit anti-BPTF/FALZ antibody	Abcam	Cat. ab72036
Rabbit anti-MSL-2 antibody	Mitzi Kuroda	Kelley *et al.* (1995)
Chemicals, Peptides and Recombinant Proteins		
Formaldehyde	Polysciences	Cat. 18814-10
Vectashield with DAPI	Vector Laboratories	Cat. H-1200-10
Experimental Models: Organisms/Strains		
*H. sapiens: HeLa CCL-2*	ATCC	RRID: CVCL_0030
*D. melanogaster: Nurf301^2^*	Own laboratory	Badenhorst *et al.* (2002) FlyBase FBal0145005
*D. melanogaster: w^1118^*	Bloomington Drosophila Stock Center	RRID: BDSC_5905
*D. melanogaster: Iswi^1^*	John Tamkun	Deuring *et al.* (2000) FlyBase FBal0117726
*D. melanogaster: Iswi^2^*	John Tamkun	Deuring *et al.* (2000) FlyBase FBal0117725
